# Prognostic factors and outcomes of nonseminomatous germ cell tumours of testis—experience from a tertiary cancer centre in India

**DOI:** 10.3332/ecancer.2020.1145

**Published:** 2020-11-18

**Authors:** Lekha Madhavan Nair, K M Jagathnath Krishna, Aswin Kumar, Susan Mathews, John Joseph, Francis Vadakkumparambil James

**Affiliations:** 1 Genitourinary Clinic, RCC, Thiruvananthapuram 695011, India; 2 Department of Epidemiology and Biostatistics, RCC, Thiruvananthapuram 695011, India

**Keywords:** nonseminoma, germ cell tumours, testis, survival

## Abstract

Germ cell tumour of the testis is the most common cancer in young men in the western world. India has the lowest incidence globally, and hence Indian data are sparse. We report the outcomes of patients with nonseminomatous germ cell tumours of testis treated at a tertiary cancer centre in South India over a period of 10 years. Patients with a histopathological diagnosis of nonseminomatous germ cell tumours of the testis from 1 January 2006 to 31 December 2016 were included in the study. Patient demographics, tumour characteristics and treatment details were retrieved from case records. Kaplan–Meier method was used to estimate progression-free survival (PFS) and overall survival (OS). Cox regression model was used to analyse the prognostic factors.

One hundred and nineteen patients with nonseminomatous germ cell tumours of the testis were included in the study. The median follow-up was 81 months. The estimated 4-year OS and progression-free survival were 87.1% and 84.5%, respectively. The four-year OS for good, intermediate and poor-risk groups was 93.6%, 87.5% and 52.6%, respectively. The PFS at 4 years was 91.4%, 87.8% and 47.4% for good, intermediate and poor-risk groups, respectively. The presence of nonpulmonary visceral metastasis and biochemical response after chemotherapy were significant predictors for OS and PFS in multivariate cox proportional hazards regression. The survival figures are comparable to the rest of the world except in the poor prognostic risk group. The inferior survival noticed in this group of patients may be due to the lack of good salvage procedures. High-dose chemotherapy with stem-cell support may be considered more often for this group of patients.

## Introduction

Testicular cancer accounts for 1%–2% of all malignancies in men. Ninety-five per cent of all testicular cancers are germ cell tumours [1]. Among these, about 50% are nonseminomatous germ cell tumours (NSGCT). It is the most common cancer in young men in the western world. The age-standardised incidence rate of testicular cancer in India is 0.5 per 100,000 population, while it is 6.7 and 5.6 per 100,000 population for Europe and the United States, respectively [2]. India has the lowest incidence globally, and hence Indian data on testicular germ cell tumours are sparse [3–5]. Here, we report the outcomes of patients with NSGCT of testis treated at a tertiary cancer centre in South India over a period of 10 years.

## Patients and methods

This retrospective study was conducted after getting approval from the Institutional Review Board. Patients with a histopathological diagnosis of nonseminomatous germ cell tumours of the testis from 1 January 2006 to 31 December 2016 were included in the study. Patients with age ≤15 years were excluded from the study as they were treated in the Paediatric Oncology Department.

Patient demographics, tumour characteristics, stage of the disease, pre- and postorchidectomy tumour markers, histopathological features and treatment details were retrieved from case records. Patients were staged according to the UICC 7th edition TNM staging (Tumour, Node, Metastasis). For uniformity of reporting, patients from 2006 to 2009 were retrospectively staged according to the UICC 7th edition. Computed Tomography (CT) scan of thorax, abdomen, pelvis and serum tumour markers, namely, Alfa Feto Protein (AFP), β-human chorionic gonadotropin (HCG) and lactate dehydrogenase (LDH), were used for staging. Postorchidectomy tumour markers were estimated for the International Germ Cell Cancer Collaborative Group (IGCCCG) risk grouping [6].

### Treatment

All patients underwent orchidectomy. One hundred and eleven patients underwent high-inguinal orchidectomy, and eight patients underwent scrotal orchidectomy. All stage IA and IB patients were given the options of chemotherapy versus active surveillance. Those patients who were willing to adhere to surveillance protocol were kept under active surveillance. Patients with stage IS disease received two or three cycles of BEP chemotherapy (Bleomycin 30 Units IV in 50 mL Normal Saline over 15 minutes on days 1, 8 and 15, Etoposide 100 mg/m^2^/day IV infusion in 500ml Normal Saline on days 1 to 5, Cisplatin 20 mg/m^2^/day IV in 100 mL Normal Saline over 30 minutes on days 1 to 5, repeated every 21 days). Stage II and III patients were treated according to the IGCCCG risk group. Good-risk patients received three cycles of BEP or four cycles of EP (Etoposide 100 mg/m^2^/day IV infusion in 500 mL Normal Saline on days 1–5, Cisplatin 20 mg/m^2^/day IV in 100mL Normal Saline over 30 minutes on days 1–5, every 21 days). Intermediate- and poor-risk group received four cycles of BEP or four cycles of VIP (Etoposide 75 mg/m2 IV infusion on days 1–5, Ifosfamide 1.2 g/m2 IV infusion on days 1–5, Cisplatin 20 mg/m2 IV infusion on days 1–5, Mesna 400 mg/m2 IV infused over 30 min before ifosfamide, then at 4 and 8 hours after the start of each ifosfamide dose, repeated every 21 days). The response was assessed with serial CT scans and tumour markers. Serum tumour markers, namely, AFP, β-HCG and LDH, were monitored every 3 weeks during chemotherapy and were repeated after 1 month of completion of chemotherapy. CT scan was done after 1 month of completion of chemotherapy in patients with a biochemical response. The radiological response was measured using RECIST criteria version 1.0. Patients in radiological and biochemical remission were kept on follow-up. Patients with normal serum tumour marker levels, but with residual retroperitoneal nodes more than 1 cm short-axis diameter, were considered for RPLND. Second-line chemotherapy was given for those with raised tumour markers.

### Follow-up

Patients were followed up for 3 months with tumour markers and clinical examination during the first two years, 6 months up to 5 years and 1 year after that. Imaging was done every six months for two years. Patients on active surveillance were followed up according to the surveillance protocol. Clinical examination, serum tumour markers and chest X-ray were done every 2 months during the first year, every 3 months during the second year, every 4 months during the third year and every 6 months for years 4 and 5. CT of thorax, abdomen and pelvis was done at 3 months, 12 months, 24 months and 36 months.

### Statistical methods

Patient and tumour characteristics were analysed using descriptive statistics. Kaplan–Meier method was used to estimate progression-free survival (PFS) and overall survival (OS) and compared using the log-rank test. OS was calculated from the date of diagnosis to the date of death or last follow-up. PFS was computed from the date of diagnosis to the date of relapse or progression of disease or death. Cox regression model was used to analyse the prognostic factors. Statistical analysis was done using SPSS software version 11.0.

## Results

One hundred and thirty patients were registered with a diagnosis of nonseminomatous germ cell tumours of the testis from 1 January 2006 to 31 December 2016. Among these, five patients came for a second opinion. Four patients abandoned the planned treatment, and one patient died before starting treatment. Only 119 patients completed the planned treatment and were included for final analysis. None of the patients was lost to follow-up. Follow-up was updated using clinical data and telephonic information. The median age at diagnosis was 27 years. The median tumour size was 6.0 cm. Patient characteristics are summarised in Table 1.

One hundred eighteen patients underwent primary orchidectomy, while one patient had the surgery after completion of chemotherapy. Fifteen patients had a scrotal violation of which eight had scrotal orchidectomy while seven patients had prior trans-scrotal biopsy/aspiration. Three patients with stage IA disease were kept on active surveillance after orchidectomy. Patients who were considered unreliable for regular follow-up were given adjuvant chemotherapy. Two cycles of BEP were given for 22 patients with stage IA/IB. One course of BEP was given for a patient with stage IA disease. All patients with stage IS disease received two or three cycles of BEP. Among the stage II and III good risk patients, 27 received three cycles of BEP and one patient was treated with four cycles of EP. EP was given due to the non-availability of bleomycin during that time. Forty-four patients received four cycles of BEP for intermediate or poor-risk disease , and one patient was treated with our cycles of VIP (Bleomycin was avoided due to the presence of extensive bilateral lung metastases and a past history of smoking). Grade 3 or 4 neutropenia was documented in 12 patients. Eight patients developed bleomycin induced lung toxicity and were managed with oral prednisolone.

### Treatment response and relapse

Among the 116 patients who underwent chemotherapy,106 achieved biochemical remission and 69 patients attained complete radiological remission. Twelve patients with residual radiological abnormality were kept on follow-up as there was a serial decrease in tumour size in the follow-up CT scans. Postchemotherapy retroperitoneal lymph node dissection (RPLND) was carried out for 14 patients with residual paraaortic nodes: five patients had mature teratoma; five had no viable tumour; four had viable tumour cells in the pathology specimen. Those with viable tumour cells on RPLND were given two more cycles of chemotherapy with VIP regimen. Patients with residual disease at multiple sites were not operated either due to nonacceptance by the patients or due to the increased risk of surgical complications. Six patients progressed after first-line chemotherapy and received second-line chemotherapy. Four of them received VIP (Etoposide, Ifosfamide, cisplatin), one patient received TIP (paclitaxel, ifosfaide, cisplatin) regimen and VeIP (vinblastine, ifosfamide, cisplatin) was given for another patient as the second line. All the six patients progressed on second-line chemotherapy and eventually succumbed to the disease. Relapse was documented in four patients and lung was the most common site of relapse. Salvage chemotherapy was given for these patients, the regimens being VIP and TIP. Five patients developed brain metastasis, four received whole-brain radiotherapy (doses ranging from 30 Gy in 15 fractions to 40 Gy in 20 fractions), but none of them survived. The three patients who were kept on active surveillance did not show any evidence of recurrence. One patient developed a second primary malignancy – papillary carcinoma of the thyroid and another patient who received two cycles of BEP developed acute myeloid leukaemia after 1 year.

### Survival

**Figure 1.**
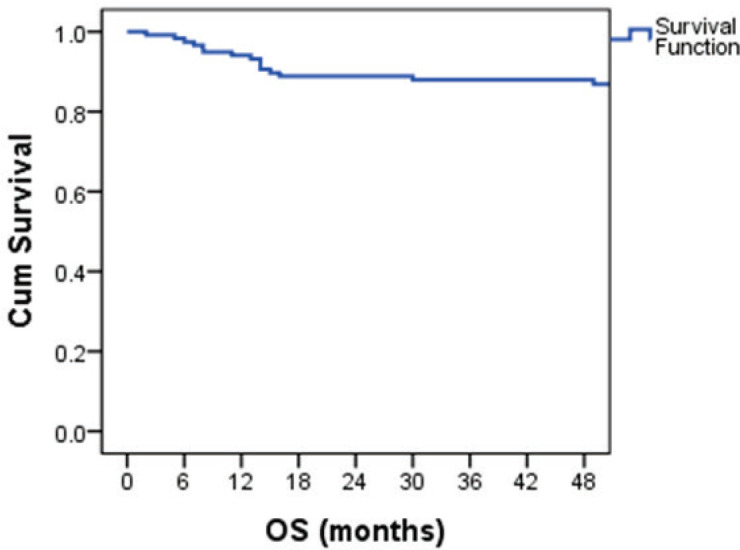
Kaplan–Meier curve showing OS probability.

The median follow-up was 81 months (5 to 161 months). The estimated 4-year OS was 87.1% (Figure 1). Overall survival was 100% for stage IA and IB, 95% for IS,86.7% for stage II and 78.5% for stage III disease. The 4-year OS for good, intermediate and the poor-risk groups were 93.6%,87.5% and 52.6% respectively. No difference in survival was noticed for cryptorchidism, scrotal violation, presence of lymphovascular space invasion (LVSI), presence of seminomatous elements, yolk sac elements, choriocarcinoma components, presence of embryonal carcinoma in the surgical specimen or T stage. There was a significant difference in survival with respect to N stage, S stage, M stage and IGCCCG risk groups. Table 2 shows the differences in overall survival according to various groups.

**Figure 2.**
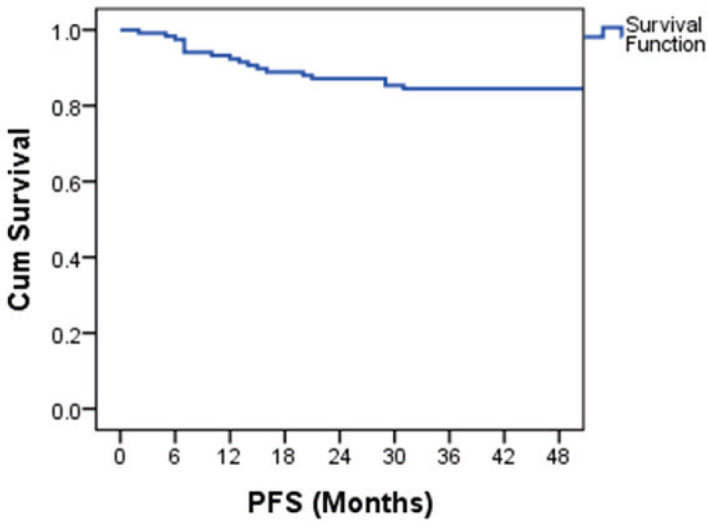
Kaplan–Meier curve showing PFS probability.

The PFS probability was 84.5% at 48 months (Figure 2). There was no difference in PFS in the presence of a history of cryptorchidism, scrotal violation, presence of LVSI, seminomatous elements, yolk sac components, choriocarcinoma or embryonal carcinoma in the resected specimen or based on T stage. However, there was a significant difference in PFS according to N stage, M stage, S status, composite stage and IGCCCG risk group (Table 3).

In univariate Cox proportional hazards regression, bHCG values, LDH levels, nodal status, presence of metastases, S stage, composite stage, IGCCCG risk group and biochemical and radiological response after first-line chemotherapy were significant predictors of survival (Table 4). In multivariate analysis, M stage and biochemical response after first-line chemotherapy remained significant factors for survival.

Serum AFP and bHCG levels, LDH values, N stage, M stage, S stage, composite stage, IGCCCG risk group, biochemical and radiological response after first-line chemotherapy were significant factors for PFS in univariate Cox proportional hazards regression. M stage, biochemical and radiological response after chemotherapy remained significant on multivariate analysis also.

## Discussion

The incidence of testicular germ cell tumours is low in the Indian population. There is a paucity of Indian data on the epidemiology and treatment outcomes of this rare cancer. Hence, we conducted this retrospective analysis of nonseminomatous germ cell tumours of testis treated at a Regional Cancer Centre in South India.

The median age at presentation was 27 years (17–53 years) which is similar to that reported in the literature [7], though a few studies have reported a median age above 30 years [8, 9]. Cryptorchidism is associated with an increased risk of testicular germ cell tumours [10]. In our series, seven patients (5.8%) developed the disease in the undescended testis; five of them were surgically corrected during childhood. The incidence is less when compared to previously published Indian data, where 12.5% of tumours developed in the undescended testis [11]. The incidence of undescended testis in our part of the world is probably decreasing, as seen from our previous publications [12, 13]. Presence of LVSI is considered as an independent prognostic factor for recurrence in stage I nonseminomatous tumours [14], but due to the limited data on LVSI, it was not assessed in this study.

About half of the patients in our series presented with stage III disease. Other reported series from India has published a similar proportion of patients with NSGCT in advanced stages [3–5]. Only 27% of patients presented with stage I (IA and IB) disease. Of these, only three patients were placed on active surveillance which is very less compared to the western literature [15, 16]. This may be due to the poor compliance noticed in our young male population. Even though RPLND is an accepted adjuvant treatment for stage I NSGCT, none of our patients underwent primary RPLND.

Among patients with metastatic disease, 38.3% had good risk disease and 26.02% of patients were in the poor-risk category. This is similar to most of the published literature where the majority of the patients were categorised as good-risk group [17, 18]. The proportion of poor-risk patients is comparable to previously published Indian data also [3, 4].

Biochemical complete response was seen in 89% of patients after first-line chemotherapy. However, only 58% of patients achieved a complete radiological response. Thirty-nine patients had the residual paraaortic nodal disease after first-line chemotherapy, but only 14 patients underwent RPLND. The rates of post-chemotherapy RPLND were less in our series compared to some previously published data [18, 19]. Many of our patients refused surgery, considering the likely complications such as retrograde ejaculation and the need for major vascular repair.

Ten patients received second-line chemotherapy on recurrence or progression, the regimens being Paclitaxel, Ifosfamide and Cisplatin (TIP), Etoposide, Ifosfamide and Cisplatin (VIP) and Vinblastine, Ifosfamide and Cisplatin (VeIP). Several salvage chemotherapy regimens have been tried in tumours refractory to BEP and TIP [20, 21]. High-dose chemotherapy and stem-cell transplantation are considered an effective option for relapsed germ cell tumours [22, 23]. In this study, none of the patients was salvaged with high-dose chemotherapy and stem-cell transplantation.

The four-year OS and PFS were 87.1% and 84.5%, respectively. Survival was 100% for stage 1 patients. The four-year survival for IS, II and III were 95%, 86.7% and 78.5%, respectively. The survival rates are better compared to the previously published Indian data [4, 5]. Among the patients with metastatic disease, four-year OS was 93.6%,87.5% and for 52.6% for good, intermediate and poor-risk groups, respectively. The PFS at four years was 91.4%,87.8% and 47.4% for good-, intermediate- and poor-risk NSGCT, respectively. These figures are comparable to the data published by IGCCCG [6]. Due to the advances in chemotherapy and stem-cell transplantation in the salvage setting, the outcomes of poor-risk patients have improved over the years. The five-year survival has improved up to 70% in some studies [7, 17, 19]. The 5-year PFS ranges from 54% to 58% in those studies [7, 17]. However, our salvage options were limited, and hence the survival of poor-risk patients remains low.

Age greater than 40 years was found to be associated with increased mortality in germ cell tumours [16, 17]. However, in our study, age was not related to outcome. Only nine patients were aged >40 years, and that may be the reason for the nonsignificant association. Cryptorchidism and scrotal violation had no association with survival in the present study similar to other studies [4]. Presence of embryonal carcinoma and LVSI was thought to be associated with increased risk of recurrence in stage I NSGCT [14]. We could not find any association of embryonal carcinoma with PFS or OS, and LVSI was not assessed due to incomplete data. Presence of seminoma, choriocarcinoma and yolk sac elements did not influence survival in this study similar to the study reported by Heinzelbecker *et al* [24].

Many studies have established the role of serum biomarkers in predicting response and survival [6, 25, 26]. In univariate analysis, serum tumour marker levels, namely, AFP, beta HCG, LDH values, were significant predictors of OS and LDH, BHCG values for PFS. An increasing nodal size was reported as a negative prognostic factor in many studies [4, 26]. N stage was found to be a significant predictor for OS and PFS in this study as well.

Nonpulmonary visceral metastasis has been established as a negative prognostic factor for survival in many studies [6, 19]. This finding was confirmed in this study also. The most important prognostic factor reported in the majority of the studies was risk group [4, 6]. The patients in the poor- and intermediate-risk groups had a higher risk of progression or death compared to those in the good-risk group.

The limitations of the study are its retrospective nature, the limited data on LVSI and chemotherapy toxicities as well as lack of information on fertility issues after treatment.

Majority of the testicular cancers are diagnosed at an advanced stage in India. Early detection can be achieved with awareness programmes for both general practitioners and the public. High-risk patients should be referred to specialised high-volume centres to improve outcomes. All these high-volume centres should be equipped with provision for high-dose chemotherapy and stem-cell transplantation.

## Conclusion

This is the second-largest retrospective series on NSGCT from India with good follow-up information. The survival figures are comparable to the rest of the world except in the poor prognostic risk group. The inferior survival noticed in this group of patients may be due to the lack of good salvage procedures, which is mainly due to the limitation of resources. Hence high-dose chemotherapy with stem-cell support may be considered more often for this group of patients.

## Source of funding

None.

## Conflicts of interest

None.

## Figures and Tables

**Table 1.** Patient characteristics.

Parameters	Number (percentage)
Age(years) <40 ≥40	110 (92.43) 9 (7.56)
Cryptorchidism Yes No	7 (5.82) 112 (94.11)
Laterality Right Left	60 (50.42) 59 ((49.57)
Scrotal violation Yes No	15 (12.6) 104 (87.39)
Seminomatous elements Yolk sac components Embryonal carcinoma components Choriocarcinoma elements	31 (26.1) 72 (60.5) 68 (57.1) 15 (12.6)
LVSIYes No Status unknown	16 (13.44) 99 (83.19) 4 (3.36)
T stage T1 T2 T3 T4	95 (79.83) 14 (11.76) 8 (6.72) 2 (1.68)
N stage N0 N1 N2 N3	55 (46.21) 15 (12.60) 27 (22.68) 22 (18.48)
M stage M0 M1a M1b	78 (65.54) 30 (25.21) 11 (9.24)
S stage S0 S1 S2 S3	33 (27.73) 43 (36.13) 30 (25.21) 13 (10.92)
Composite stage I IS II III	26 (21.84) 20 (16.80) 15 (12.60) 58 (48.73)
Risk group Good Intermediate Poor	48 (51.61) 26 (27.95) 19 (20.43)

**Table 2.** Overall survival probability according to various groups.

Groups	OS at 48 months (%)	Standard error (%)	*p*-value
N0 N1 N2 N3	96.3 92.9 84.0 63.6	2.5 6.9 7.3 10.3	0.001
M0 M1a M1b	92.1 85.8 54.5	3.1 6.6 15	0.001
S0 S1 S2 S3	100 92.7 83.0 46.2	- 4.1 6.9 13.8	0.001
Stage 1 Stage 1S Stage 2 Stage 3	100 95 86.7 78.5	- 4.9 8.8 5.5	0.012
Good risk Intermediate risk Poor risk	93.6 87.5 52.6	3.6 6.8 11.5	0.001

**Table 3.** Progression free survival probability according to various groups.

Groups	PFS at 48 months (%)	Standard error (%)	*p* value
N stage N0 N1 N2 N3	92.6 92.9 79.8 63.6	3.5 6.9 8.1 10.3	0.001
M stage M0 M1a M1b	90.8 82.1 45.5	3.3 7.3 15	0.001
S stage S0 S1 S2 S3	96.9 90.2 82.6 46.2	3.1 4.7 7.1 13.8	0.001
Composite stage I IS II III	96.0 95.0 86.7 74.8	3.9 4.9 8.8 5.8	0.022
IGCCCG risk group Good Intermediate Poor	91.4 87.8 47.4	4.1 6.6 11.5	0.001

**Table 4.** Cox proportional hazard regression model on prognostic factors for OS and PFS.

	OS				PFS			
Factors	Hazard ratio (HR)	95% confidence interval		*p* value	HR	95% CI		*p* value
		Lower	upper					
Age (≥40 versus <40)	1.781	0.407	7.790	0.443	1.562	0.361	6.761	0.551
Cryptorchidism (yes versus no)	1.086	0.144	8.187	0.937	1.213	0.162	9.091	0.851
Scrotal violation (yes versus no)	25.46	0.068	9560	0.284	2.986	0.398	22.37	0.287
Seminoma components (no versus yes)	1.302	0.458	3.697	0.620	1.439	0.547	3.788	0.461
Yolk sac elements (no versus yes)	0.738	0.285	1.912	0.531	0.902	0.363	2.242	0.824
Choriocarcinoma (no versus yes)	0.410	0.054	3.094	0.387	0.365	0.049	2.732	0.326
Embryonal carcinoma (no versus yes)	0.669	0.258	1.733	0.408	0.672	0.273	1.655	0.387
AFP values^a^ A1 versus A0 A2 versus A0 A3 versus A0	1.066 3.326 3.764	0.325 1.013 0.452	3.495 10.919 31.33	0.137 0.915 0.048 0.220	0.916 3.372 3.210	0.291 1.131 0.394	2.887 10.05 26.13	0.077 0.881 0.029 0.276
bHCG values^b^ H1 versusH0 H2 versus H0 H3 versus H0	2.280 0 10.586	0.766 - 2.974	6.787 - 37.67	0.004 0.139 0.985 0.001	1.729 0 8.105	0.627 - 2.429	4.771 - 27.04	0.009 0.290 0.983 0.001
LDH values^c^ L1 versus L0 L2 versus L0 L3 versus L0	2.214 4.584 18.427	0.405 1.379 4.099	12.087 15.235 82.832	0.002 0.359 0.013 0.001	1.778 4.124 14.850	0.345 1.381 3.530	9.166 12.31 62.47	0.002 0.491 0.011 0.001
T stage T2 versus T1 T3 versus T1 T4 versus T1	2.482 0.991 0	0.800 0.129 -	7.690 7.620 -	0.470 0.115 0.993 0.987	2.141 0.840 0	0.705 0.110 -	6.506 6.387 -	0.590 0.179 0.866 0.986
N stage N1 versus N0 N2 versus N0 N3 versus N0	1.258 3.017 9.892	0.131 0.675 2.672	12.098 13.492 36.618	0.002 0.842 0.148 0.001	0.934 2.882 7.429	0.104 0.773 2.284	8.355 10.74 24.16	0.004 0.951 0.115 0.001
M stage M1a versus M0 M1b versus M0	2.882 9.152	0.929 2.784	8.940 30.00	0.001 0.067 0.001	2.483 9.276	0.834 3.108	7.393 27.67	0.001 0.102 0.001
S stage S2 versus S1 S3 versus S1	2.307 7.800	0.650 2.279	8.181 26.695	0.003 0.196 0.001	2.664 7.892	0.779 2.306	9.108 27.01	0.004 0.118 0.001
Composite stage Stage II versus I Stage III versus I	6.257 13.119	0.561 1.724	69.033 99.832	0.034 0.134 0.013	3.172 7.052	0.447 1.611	22.52 30.86	0.026 0.248 0.010
Risk group Intermediate versus good Poor versus good	2.752 10.194	0.615 2.754	12.316 37.737	0.001 0.185 0.001	2.043 8.512	0.510 2.664	8.179 27.20	0.001 0.313 0.001
Radiological complete response after 1st line chemo (no versus yes)	13.558	3.097	59.352	0.001	9.803	2.853	33.68	0.001
Biochemical complete response after 1st line chemo (no versus yes)	16.913	6.438	44.433	0.001	14.282	5.636	36.19	0.001

a A0- normal AFP values, A1-AFP < 1,000 ng/mL, A2-AFP ≥1,000 and ≤10,000 ng/mL, A3-AFP > 10,000 ng/mL

b H0-normal bHCG values, H1-bHCG < 5,000 iu/L, H2-bHCG > 5000 iu/L ≤50,000 iu/L, H3-bHCG > 50,000 iu/L

c L0-normal LDH values, L1-LDH <1.5× upper limit of normal, L2-LDH ≥ 1.5× upper limit of normal and ≤10× upper limit of normal, L3-LDH > 10× upper limit of normal
